# Hindfoot Nailing for Surgical Management of Open Ankle Fractures in the Elderly: A Systematic Review

**DOI:** 10.7759/cureus.75838

**Published:** 2024-12-16

**Authors:** Aditya V Boppana, Akash S Boppana, Michael A Roberts, Christopher J Wall

**Affiliations:** 1 Orthopedics and Traumatology, Queensland Health, Toowoomba, AUS; 2 General Surgery, Univeristy of Auckland, Auckland, NZL; 3 Orthopedics, Queensland Health, Toowoomba, AUS

**Keywords:** arthrodesis, elderly, hindfoot nails, morbidity, mortality, open ankle fractures, systematic review

## Abstract

Open ankle fractures in the elderly are increasingly common, with significant morbidity and mortality. Management is challenging due to poor soft tissue conditions, comorbidities, and limited functional independence. While traditional surgical options include external fixation or open reduction and internal fixation (ORIF), hindfoot nail (HFN) fixation may offer advantages, including immediate weight-bearing and reduced immobilisation complications. However, no systematic review has assessed the outcomes of HFNs in managing open ankle fractures in this population.

A systematic review was conducted in accordance with the Preferred Reporting Items for Systematic Reviews and Meta-Analyses (PRISMA) 2020 guidelines. Comprehensive searches of the Medline, CINAHL, Embase, and Cochrane databases were conducted on September 24, 2024, with inclusion criteria focused on studies involving HFN for open ankle fractures in patients aged ≥60 years. Outcomes included union rates, infection, complications, and functional recovery. Two reviewers independently performed data extraction and quality assessment (using the Newcastle-Ottawa Scale), with narrative synthesis due to study heterogeneity.

Five retrospective studies were included where open ankle fractures were treated with HFNs. Across studies, immediate post-operative weight-bearing was allowed in most cases. Union rates ranged from 85% to 100%. Infection rates varied between 6.3% and 17.9%, with implant-related complications noted in 18.7% of cases. Functional outcomes, where reported, were moderate, with scores such as the Olerud-Molander Ankle Score (OMAS) averaging 45-57 (62 preoperatively). Mortality at six to 12 months ranged from 15% to 61%. Compared to ORIF, HFNs demonstrated no difference in union rates or complications, including deep vein thrombosis (DVT) and pulmonary embolism (PE). HFNs utilised in arthrodesis resulted in increased wound complications, and implant removal reoperations compared to ORIF (10%-20% vs. 5%-10%) in frail patients with compromised soft tissues. HFNs used for fixation only resulted in similar complication rates to ORIF.

HFNs appear to be a viable option for managing open ankle fractures in elderly patients. Offering advantages such as immediate weight-bearing and reduced immobilization risks. However, higher rates of implant-related complications and variable functional outcomes warrant caution. Prospective, comparative studies are needed to better delineate the role of HFNs in this complex patient population.

## Introduction and background

Ankle fractures are the third most common fractures in elderly patients aged 60 years or older and are increasing in incidence, especially open ankle fractures [1]. Open ankle fractures in the elderly cause significant morbidity and mortality [2]. These are challenging to manage because of the poor soft tissue envelope, comorbidities and limited functional independence [3]. Mortality can be as high as 12% at the one-year mark [4]. 

Surgical management of open ankle fractures traditionally involved debridement and external fixation or open reduction and internal fixation (ORIF) with plates and screws [5]. The post-operative period necessitates a non-weight-bearing period of at least six weeks [5]. Complications that arise include loosening of fixation, deep vein thrombosis (DVT), pneumonia, surgical site infections, pressure ulcers and deconditioning [6]. 

Recent literature has described using hindfoot nail (HFN) fixation in the elderly population to primarily address ankle fractures, especially in cases with severe soft tissue disruption around the planned approach [7]. The proposed benefits include fracture stability and day one post-operative weight-bearing, mitigating the risks of prolonged immobilisation [5-6]. The purpose of this study was to review the current literature on managing open ankle fractures with HFNs as primary fixation in the elderly. To date, there have been no systematic reviews published on this topic. 

## Review

Methodology

This systematic review was conducted following the Preferred Reporting Items for Systematic Reviews and Meta-Analyses (PRISMA) 2020 guidelines. The protocol was registered in the PROSPERO database (registration number: CRD42021234567).

Search strategy

A structured search of the Medline, CINAHL, Embase and Cochrane databases was conducted on 24 September 2024. The search was carried out in consultation with a medical librarian at Darling Downs Health, Queensland, Australia.

The search terms are highlighted in Appendices A-C. Boolean operators were utilised as appropriate.

Study selection criteria

The inclusion criteria consisted of cohort studies, case series, randomised controlled trials, reviews and meta-analyses where HFNs were utilised as primary fixation in acute open ankle fractures that directly addressed and/or included the elderly (aged 60 years or older) as a part of the study population. These articles must have been conducted on humans, published in English from the year 2000 and needed to have a sample size of 10 patients or greater with open ankle fractures. 

The exclusion criteria included studies utilising HFNs in other disorders apart from open ankle fractures; studies with a sample size of less than 10 patients; case reports, textbook chapters, letters, conference abstracts or editorials; study population aged less than 60 years of age and studies published earlier than 2000. 

Screening and selection process****

The initial search yielded 3,417 total studies. First deduplication was performed, which reduced the number to 2,771 studies. Limiting the literature to year and study type with additional manual deduplication gave a final number of 1,978 studies. 

Abstracts were screened via two independent reviewers (AVRB and ASB) using the JBI Summari software in relation to the inclusion and exclusion criteria. When sufficient information from the abstract was not present to determine suitability for inclusion, the full paper was retrieved. Reviewers were blinded to each other’s decisions. Any discrepancies in included studies from the initial abstract screen were discussed and resolved with a third independent reviewer (MAR). 

Full studies selected for review underwent further blinded screening by the same two reviewers. Again, a third independent reviewer was consulted to address any discrepancies. 

**Figure 1:**
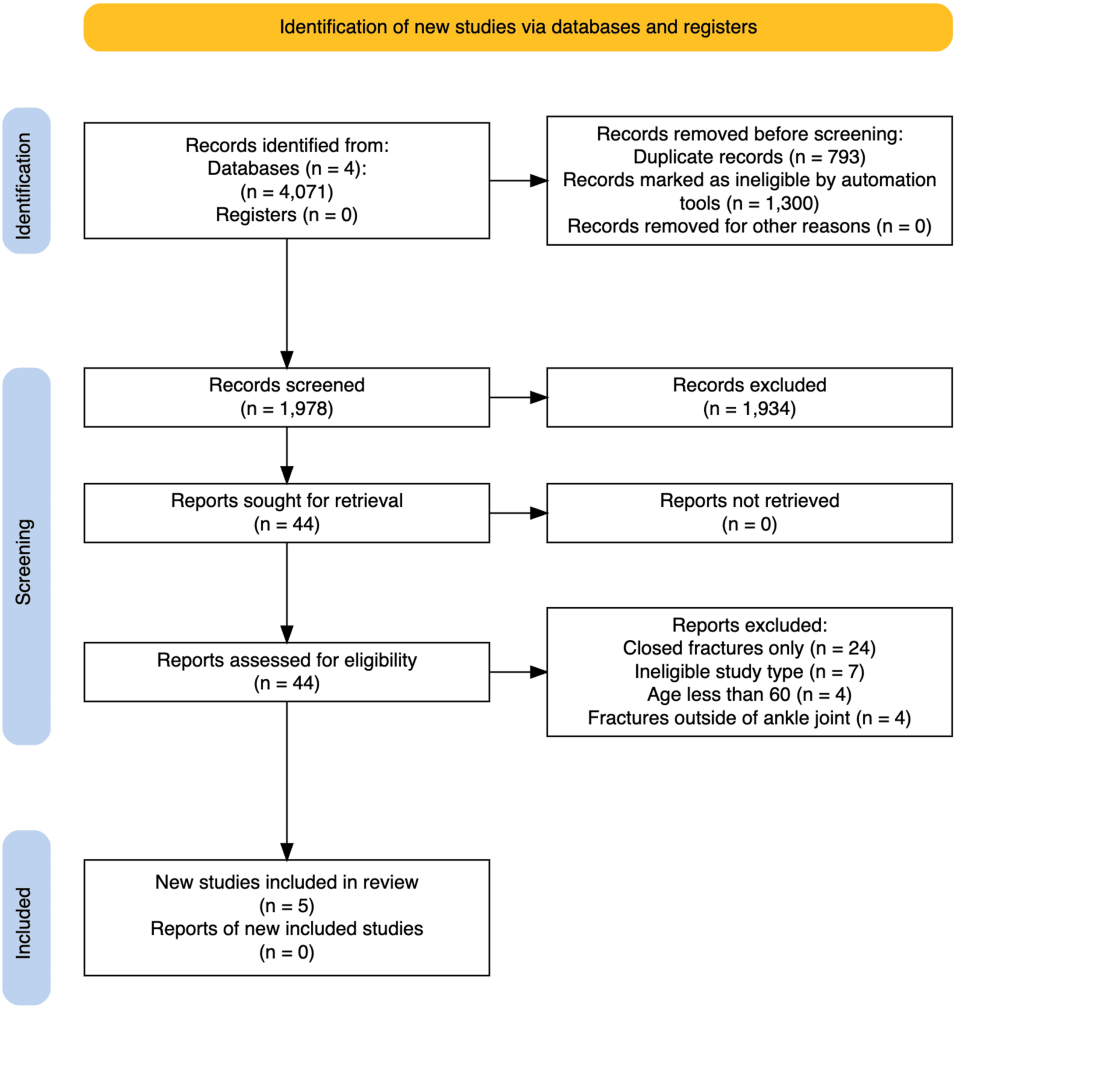
PRISMA diagram showing the study selection process. PRISMA, Preferred Reporting Items for Systematic Reviews and Meta-Analyses

A PRISMA flow diagram is shown in Figure 1 [8]. 

Data collection process

Data were extracted independently by two reviewers using a standardised form on an Excel spreadsheet. Extracted data included study characteristics, patient demographics, ankle fracture type (bimalleolar, trimalleolar, Danis-Weber classification), severity of open fracture (Gustilo-Anderson classification), surgical technique (prepared vs. unprepared joint surfaces), implant details, union rates, follow-up period, infection rate, non-union, implant failure, revision surgery, other complications and functional outcomes. These data points were also extracted for any comparator groups that were present within the studies. 

Risk-of-bias assessment

The Newcastle-Ottawa Scale (NOS) was used to assess the quality of non-randomised studies [9]. Studies were scored on selection, comparability and outcome assessment. The risk of bias was considered low, moderate or high based on the total score. 

Synthesis of results

Data were synthesized narratively due to heterogeneity in study designs and outcomes. A meta-analysis was not performed due to the lack of homogeneity in the included studies. 

Study characteristics and risk of bias

A total of five studies were included. All were retrospective observational studies. Appendix D highlights a summary of risk assessment for included studies. 

Study 1: Epidemiology & Management of Complex Ankle Fractures in the United Kingdom: A Multicentre Cohort Study

In 2024, Stringfellow et al. published a 57-centre retrospective observational study from the United Kingdom that addressed ORIF, external fixation and HFNs for all complex ankle fractures that occurred from 1 January 2019 to 30 June 2019 [10]. A complex fracture was defined as all open/closed AO 43/44 fractures with either diabetes mellitus, peripheral neuropathy, alcoholism, cognitive impairment, rheumatoid arthritis or polytrauma [10]. The Clinical Frailty Score (CFS), American Society of Anesthesiologists (ASA) grade and pre-operative baseline mobility were recorded [10]. The mean time to follow up was 7.8 months (maximum of 18 months) [10]. The study used appropriate statistical tests to analyse outcomes. Propensity matching adjusted for confounding factors, including age, ASA score, diabetes and frailty [10].

Study 2: Fragility Fractures of the Ankle in the Frail Elderly Patient Treatment With a Long Calcaneotalotibial Nail 

Al-Nammari et al. published a retrospective single-centre study of 48 elderly patients who sustained ankle fractures from 1 January 2010 to 1 January 2013 [11]. The fractures were all low energy and deemed unstable based on the degree of displacement. The T2 retrograde femoral nailing system (Stryker, Kalamazoo, MI) was utilised [11]. ASA grade and patient comorbidities were recorded [11]. 

HFN treatment was selected for frail patients unable to manage restricted weight-bearing post-operatively and those with severely limited pre-injury mobility [11]. Exclusion criteria included those who could walk independently for over 200 m [11]. Included were patients who required a frame for short outdoor distances or were mobile only at home, as well as those with poor bone stock due to fragility fractures or severe osteopenia [11]. The procedure was performed by a consultant in 69% of cases, with a mean operating time of 55 minutes and no intra-operative complications [11]. 

Patients were followed up for six months, and recorded outcomes included mobility, weight-bearing, union, surgical site infection, further surgery, ICU admission, mortality and cause of mortality [11]. 

Study 3: Male Sex, Gustillo-Anderson Type III Open Fracture and Definitive External Fixation are Risk Factors for a Return to the OR following the Surgical Management of Geriatric Low Energy Open Ankle Fractures 

The study conducted a retrospective analysis of 113 patients aged 60 years and older who sustained low-energy open ankle or pilon fractures, treated by fellowship-trained trauma surgeons at two Level I trauma centres between 1 January 2007 and 1 September 2019 [12]. 

The mean age of participants was 75.2 years [12]. Patients were included based on specific criteria. High-energy injuries, delayed presentation and other specific exclusions were omitted [12]. 

Surgical techniques involved fracture stabilization using ORIF and external fixation according to AO principles in addition to HFNs with patient advancement to weight-bearing based on healing status [12]. Statistical analyses included univariate comparisons and multivariate logistic regression to identify significant risk factors for outcomes [12]. 

Study 4: The Outcomes of the Management of Complex Distal Tibia and Ankle Fractures in Elderly With Tibiotalocalcaneal Nails in a Minimum 12-Month Follow-Up Period 

Kotsarinis et al. conducted a retrospective case series to evaluate the use and effectiveness of the Phoenix Ankle Arthrodesis Nail System (Zimmer Biomet, Warsaw, IN) in treating distal tibia and ankle fractures in elderly patients [13]. The study was conducted over a period from January 2013 to December 2020 [13]. Minimum follow-up time was at least 12 months [13]. 

The inclusion criteria consisted of elderly patients with distal tibia and ankle fractures treated with the Phoenix Ankle Arthrodesis Nail [13]. Chronic issues, such as malunions and non-unions, as well as patients who died or were lost to follow-up, were excluded [13]. 

Primary outcomes included union, time to union, surgical site infections, infection of implants, further surgery, removal of implants, implant complications and functional outcomes assessed via the Olerud-Molander Ankle Score (OMAS) [13]. 

Study 5: The Use of Hindfoot Nails for Elderly Complex Distal Tibial and Ankle Fractures

This retrospective observational review was conducted by Carin et al. at a Level I adult trauma centre and examined outcomes of 70 elderly patients (aged 65-98 years) who underwent HFN fixation for ankle or distal tibial fractures at a trauma centre in southwest England between December 2013 and August 2018 [14]. The study focused on patients over 65 years old, with data collected on injury type, ASA grade, complications, and further treatments [14]. The primary outcome was reoperation, with secondary outcomes including infection rates, hardware failure, removal of implants and one-year mortality [14]. 

Three types of HFNs were used: the T2 Ankle Arthrodesis Nail, the Versanail, and the Titanium Cannulated Hindfoot Arthrodesis Nail, with nail lengths selected to minimise the risk of periprosthetic fractures [14]. 

Open fractures were managed with either single or staged surgeries [14]. Follow-up continued for at least 12 months, with reoperation rates and infection guided by the Anti-Infection Global Expert Committee’s criteria for fracture-related infection (FRI) [14]. 

Results

Table 1 shows the results of this study.

**Table 1:** Results table showing study location, fracture characteristics, demographics, comorbidities, primary and secondary outcomes and comparator group outcomes. HFN, hindfoot nail; DVT, deep vein thrombosis; PE, pulmonary embolism; BMI, body mass index; ASA, American Society of Anesthesiologists; IHD, ischaemic heart disease; CKD, chronic kidney disease; TTC, tibiotalocalcaneal; EtOH, ethanol (alcohol); OMAS, Olerud-Molander Ankle Score; BKA, below-knee amputation

Study name	Authors and journal	Location	Year	Number of total fractures and classification	Number of open fractures and severity	Average age and demographics	Patient comorbidities	Indications for HFN	Method of fixation and post-operative protocol	Primary outcomes	Secondary Outcomes	Comparator group(s)	Comparator group outcomes
Epidemiology & Management of Complex Ankle Fractures In The United Kingdom: A Multicentre Cohort Study [10]	Stringfellow et al. Journal of Injury	United Kingdom	2024	1,145 total ankle fractures; 111 in the HFN group	285 open fractures in total;43 in HFN group; 33/43 HFN fixation; 10/43 HFN fusion	84.8 years for HFN fixation, with 40/72 (55.6%) being male; 72.03 for HFN fusion, with 13/72 (33.3%) being male	HFN group: ASA grade 1: 20/111 ASA grade 2: 28/111 ASA grade 3: 45/111 ASA grade 4: 8/111 Diabetes: 26/111 Peripheral neuropathy: 11/111 Alcoholism: 9/111 Polytrauma: 7/111 Dementia: 8/111 Mental health diagnosis: 7/111 Independent mobility: 52/72 (72.2%) for fixation, 16/39 (41%) for fusion Mobility with aid: 19/72 (16.4%) for fixation, 18/39 (46.1%) Frailty score: 2.97± 2.00 fir fixation via HFN and 4.00 ± 1.84 for fusion	No formal indications	HFN as fixation (joints not prepared); HFN for primary arthrodesis (joints prepared); 35.1% of HFN patients allowed to bear weight immediately post-op	Patient comorbidities as listed	DVT: 1/39 (2.6%) in the fusion group and 0/72 in the fixation group. PE: 2/39 (5.1%) in the fusion group and 0/72 in the fixation group. Wound infection: 7/39 (17.9%) in the fusion group and 2/72 (2.8%) in the fixation group. Wound breakdown: 8/39 (20.5%) in the fusion group and 1/72 (1.4%) in the fixation group; further procedure: 8/39 (20.5%) in the fusion group and 3/72 (4.2) in the fixation group. Removal of metal work: 7/39 (17.9%) in the fusion group and 2/72 (2.8%) in the fixation group	ORIF: ASA grade 1: 280/1073 ASA grade 2: 450/1073 ASA grade 3: 235/1073 ASA grade 4: 33/1073 Diabetes: 254/1073 Peripheral neuropathy: 26/1073 Alcoholism: 155/1073 Polytrauma: 117/1073 Dementia: 29/1073 Mental health diagnosis: 88/1073 Independent mobility: 952/1073 Mobility with aid: 112/1073 Frailty score: 2.30± 1.43 for ORIF and 2.67 ± 1.78 for extended ORIF External-Fixation: ASA grade 1: 288/1116 ASA grade 2: 471/1116 ASA grade 3: 246/1116 ASA grade 4: 34/1116 Diabetes: 268/1116 Peripheral neuropathy: 27/1116 Alcoholism: 164/1116 Polytrauma: 119/1116 Dementia: 30/1116 Mental health diagnosis: 94/1116 Independent mobility: 987/1116 Mobility with aid: 119/1116 Frailty score: 2.30± 1.43 for ORIF and 2.67 ± 1.78 for extended ORIF	ORIF DVT: 4/1068 (0.4%) PE: 6/1068 (0.6%) Wound infection: 91/1068 (8.5%) Wound breakdown: 66/1068 (6.2%) Further procedure: 94/1068 (8.8%) Removal of metal work: 89/1068 (8.3%) External - Fixation DVT: 3/84 (3.6%) PE: 2/84 (2.4%) Wound infection: 10/84 (11.9%) Wound breakdown: 1/84 (1.2%)
Male Sex, Gustillo-Anderson Type III Open Fracture and Definitive External Fixation Are Risk Factors for a Return to or Following the Surgical Management of Geriatric Low-Energy Open Ankle Fractures [11]	Fourman et al. Journal of Injury	Not mentioned	2022	113 total fractures, 95 ankle fractures and 36 HFN in total	113 fractures 4 Grade 1 53 Grade II 56 Grade III Mean skin deficit was 7.5cm ± 4.1cm	75.2 ± 9.8 years 27.4% male	Mean BMI 32.6 ± 7.9 17.7% BMI >= 40 17.7% smokers Mean age-adjusted Charlson comorbidity index was 5.5 ± 2.0	Low-demand patients (no formal definition); compromised soft tissue envelope; limb salvage; peripheral vascular disease; poor bone quality	Formal joint preparation in patients with amenable soft tissues only with no preparation of subtalar joint in any patient	90-day unplanned return to the operating theatre; 25 patients from all fixation methods; 2/25 (8%) from the HFN group; P-value 0.003, odds ratio 0.3; other statistically significant factors: Males 13/25 (52%, P-value 0.002); Gustillo Type 3 open fracture 18/25 (72%, P-value 0.01)	90-day event (mortality or readmission) 14/36 from HFN (41.2%, P value 0.2) Other statistically significant features: Surgical site infection 15/34 (44.1%, P value 0.002) Minimal ambulator pre-injury 20/34 (58.8%, P value 0.06) 1-year mortality: HFN 13/21 (61.9%, P-value 0.002)	Definitive External-fixation ORIF	90-day unplanned return to the operating theatre: Definitive Ex-Fix 7/25 ORIF 15/25 ninety-day event (mortality or readmission): Definitive Ex-Fix 4/34 ORIF 14/34 one-year mortality: Definitive Ex-Fix 3/21 (14.3%, P 0.4) ORIF 3/21 (14.3%, P 0.0001)
Fragility Fractures of the Ankle in the Frail Elderly Patient Treatment With a Long Calcaneotalotibial Nail [12]	Al-Nammari et al. The Bone and Joint Journal	United States	2014	48 total 30/48 bimalleolar 18/48 trimalleolar	19 total 10/19 patients required formal soft tissue coverage - no Gustillo Anderson classification	82 years 85% women Preinjury residence: 23% own home 42% sheltered housing 35% Nursing home	0 patients had independent mobility with an Average ASA score of 3 Average of 3.3 comorbidities per patient in the total cohort 42% IHD 25%HF 31% COPD 33% CVA/TIA 15% Malignancy 17% EtOH dependence 27% Chronic glucocorticoid usage 31% Diabetes mellitus 25% Chronic warfarin usage 13% CKD 33% Dementia 31% Liver disease 42% previous osteoporotic fracture 8% inflammatory arthropathy	Decided by consultant on call Patients considered physically or mentally frail to manage restricted weight bearing postoperatively. Patients who are non-independent ambulators (frame or walker) and can walk less than 200 m	TTC nail via Stryker T2 Femoral retrograde nailing system. No joint preparation	Six-month mortality of total cohort - 35%; 90% of the total cohort returned to the pre-operative level of function and mobility Fracture union – mean of 9 weeks. 0 cases of non-union, nail breakage or peri-prosthetic fracture 14 patients of the total cohort who were intellectually capable undertook the Olerud and Molander questionnaire: -Mean pre-operative score: 62 (40-80) -Mean pre-operative score post operatively at 6 months: 57 (35-70)	Outcomes were for the entire cohort: Returned to own: 82% of those from home returned 85% of those from sheltered housing returned back 100% of those from nursing home returned Surgical site infections - 4% Asymptomatic valgus malunion 4%; three patients had distal locking bolt failure – 2/3 patients had removal 1 amputation for a grade 3c fracture Medical complications: Pneumonia 19% Myocardial infarction 10%		
The Outcomes of the Management of Complex Distal Tibia and Ankle Fractures in Elderly With a Tibiotalocalcaneal Nail in a Minimum 12-Month Follow-Up Period [13]	Kotsarinis et al. European Journal of Orthopaedic Surgery and Traumatology	United States	2024	32 total; 22 ankle fractures	18 open fractures Type 1: 6 Type 2: 1 Type 3a: 1 Type 3b: 10	80.2 years 12 males (37.5%)	None reported in the study	Elderly (65 years or older) Unstable ankle fractures Low functional demand Multi comorbid	Phoenix ankle arthrodesis nail system (Zimmer Biomet) Mean time to definitive management was 7.4 days Skin flap: 3 cases Gracilis muscle flap: 5 cases Fasciocutaneous flap: 2 cases Immediate post-operative weight bearing in air cast boot using crutches or Zimmer frame (unless poly traumatised patient - 2/32 patients)	Time to fracture union clinically and radiographically (3/4 cortices with cortical apposition): 30/32 had fracture union 17/18 open fractures had union 1 open ankle fracture (Open 3b trimalleolar ankle fracture with HTN, HF, AF and EtOH abuse - required amputation 3 months post HFN Mean time to union was 3.9 months	Implant related complications 8/32 (25.1%) Infection before union 2/32 (6.3%) - 1 implant removal Infection after union 3/32 (9.4%) - 3 implant removals Stress risers development 1/32 (3.1%) - 1 implant removal Osteolysis around talus 2/32 (6.3%) 6/32 nail removals (18.7%) OMAS at 6 months follow up: Mean of 45 (only 9 patients intellectually able to carry out scoring)		
The Use of Hindfoot Nails for Elderly Complex Distal Tibial and Ankle Fractures Author Links Open Overlay Panel [14]	Corin et al. Journal of Foot and Ankle Surgery	United Kingdom	2023	70 total fractures; 52 ankle fractures; 37 bimalleolar fractures; 15 trimalleolar fractures	34 open ankle fractures: 24 bimalleolar and 10 trimalleolar	No average age - all patients aged over 65; 90% females (63/70)	4/70 ASA grade 1 12/70 ASA grade 2 43/70 ASA grade 3 11/70 ASA grade 4	No formal mention of indications	T2 Ankle arthrodesis nail from Stryker Versanail from DePuy ACE orthopaedics Titanium cannulated HFN from Synthes Open fractures - single surgery where possible. If debridement was needed, done on day 0 or day 1 of admission. If no flap coverage, allowed to bear weight as tolerated immediately post-op	Reoperation rates: 4/34 (11.8%) open ankle fractures had locking bolt removed for loosening 6/18 closed ankle fractures (33.33%) had removal of locking bolt for loosening 2/34 open ankle fractures had BKAs for ongoing wound issues	Infection 4/34 open ankle fractures treated with HFN (11.8%) 2/34 (5.9%) open ankle fractures had removal of HFN for infection 1-year mortality 11/70 patients with an average age of 84 years 5/11 ASA grade 4 6/11 ASA grade 3		

Discussion 

This systematic review highlights HFNs used by multiple centres to manage open ankle fractures in elderly populations, including injuries ranging from Gustilo-Anderson type 1 to type 3c [10-14]. 

Indications for HFNs

Of the five studies included in this review, three by Al-Nammari et al. [11], Fourman et al. [12] and Kotsarinis et al. [13] reported criteria for the use of HFNs in managing open ankle fractures. All three studies identified age >60 years, low functional demand, and multi-morbidity as key indications for HFNs in both closed and open ankle fractures [11-13]. Al-Nammari et al. defined low functional demand as non-independent ambulators who can walk less than 200 m and are too physically or mentally frail to manage post-operative restricted weight-bearing [11]. However, despite low functional demand being an indication, 61.3% of Stringfellow et al.’s participants receiving HFNs were independent mobilizers [10]. 

The severity of patient comorbidities was quantified in four out of five studies. Stringfellow et al. reported an average ASA score of 2.1 for patients treated with HFNs, while Al-Nammari et al. and Corin et al. reported mean ASA scores of 3 and 2.9, respectively [10-11,14]. Fourman et al. recorded a mean age-adjusted Charlson comorbidity index of 5.5 ± 2.0 [12]. 

Additional criteria for HFN use included poor soft tissue envelope, poor bone quality, limb salvage [12], and unstable ankle fractures [13]. The severity of the open injury itself did not preclude the use of HFNs; rather, the decision depended on whether extensive operative debridement and temporary fixation were required [10,12-14]. In all studies, more severe injuries typically necessitated debridement and external fixation before definitive fixation [10-14]. This aligns with existing literature, where gross contamination or large soft tissue defects are associated with higher infection rates when primary fixation is used without prior debridement and temporary stabilisation [15]. 

Surgical Implant Selection and Joint Preparation

The majority of studies used dedicated HFNs as the implant of choice, except the one by Al-Nammari et al., who used retrograde femoral nails [10-14]. HFNs can be inserted with or without preparation of the ankle and/or subtalar joint surfaces [15]. Joint preparation involves cartilage resection and achieving coplanarity, which improves the chances of arthrodesis [15]. Benefits of joint preparation include reduced pain, improved functional outcomes, reduced implant fatigue, and higher union rates, especially for deformities, neuropathic arthropathy, and arthritis [16]. Recent literature however supports the use of HFNs in elderly, low-demand patients without joint preparation [17]. 

Only two of the five studies prepared joint surfaces. Fourman et al. prepared the ankle joint in cases with amenable soft tissues but did not prepare the subtalar joint in any patients [12]. No significant difference in outcomes was reported between prepared and unprepared joints [12]. Stringfellow et al. subdivided their HFN cohort into two groups: those with formal arthrodesis (prepared joints) and those without [10]. Patients who underwent fixation without joint preparation were older and frailer (average age 84.1 years vs. 72.0 years; average frailty score 4 vs. 2.97) [10]. The fixation group exhibited fewer complications, possibly reflecting their lower functional demand [10]. The arthrodesis group experienced increased wound complications, implant failure, and metalwork removal, consistent with findings in the literature [18]. Peripheral neuropathy was more common in the arthrodesis group but was not associated with diabetes [10]. 

Outcomes

The reported outcomes across studies were heterogeneous. Only studies by Kotsarinis et al., Corin et al. and Fourman et al. specifically analysed open fractures [12-14]. Kotsarinis et al. reported a 17/18 (94.5%) union rate for open fractures, with a mean time to union of 3.9 months [13]. Ten out of 18 patients required skin grafts or flap coverage [13]. Patients were allowed immediate post-operative weight-bearing [13]. Al-Nammari et al. reported no cases of non-union and a mean union time of nine weeks for both open and closed fractures treated with calcaneotalotibial nails, consistent with union rates approaching 100% in other literature [11,19]. 

Studies reported OMAS scores of 45 to 57 post-operatively [11,13]. However, only Al-Nammari et al. reported a pre-operative score of 62 [11]. Nonetheless, the difference in scores is marginal and the clinical significance is questionable given that patients had low functional demand to begin with. 

Complication rates were addressed in all studies. Surgical site infections were reported in 4% to 44% of cases [10-14]. Stringfellow et al. highlighted an increased rate of wound complications for those who underwent fusion as opposed to fixation alone [10]. Those from the fusion group had a wound breakdown rate of 8/39 (20.5%), infection rate of 7/39 (17.9%), 8/39 (20.5%) reoperation rate, and an implant removal rate of 7/39 (17.9%) compared to rates of 1/72 (1.4%), 2/72 (2.8%), 3/72 (4.2%) and 2/72 (2.8%) in the fixation group, respectively [10]. 

The majority of reoperations and implant removals occurred due to infection [10-14]. The return to theatre rates ranged from 2/72 (4.2%) to 8/39 (20.5%) [10-14]. Stringfellow et al. reported higher rates of reoperation and implant removal in the fusion group of 8/39 (20.5%) and 7/39 (17.9%), respectively, compared to 3/72 (4.2%) and 2/72 (2.8%) in the fixation group, respectively [10]. These findings are similar to other literature [18].

The aforementioned results indicate that utilising HFN in an open ankle fracture has no increase in wound complications, infections, reoperations or implant removal compared to HFNs used in ankle fusion in a non-traumatic setting [18-19]. 

Stringfellow et al. reported DVT and PE rates of 1/111 (0.9%) and 2/111 (1.8%), respectively; however, only 35% of those who had HFNs were allowed to mobilise day 1 post-operatively, potentially confounding the DVT and PE rates [10]. 

Only Stringfellow et al. and Corin et al. had ORIF comparator groups [10,13]. The rates of infection, further procedures and removal of metalwork were within the values reported in previous literature [17-18,20]. The rate of union for ORIF in ankle fractures in the literature varies from 80% to 100% [20-21]. The infection rates for ORIF range from 8% for superficial surgical site infections to 11% for deep wound infections [19]. The rates of reoperation vary from 5% to 15%, predominantly for infection, followed by implant loosening [22-24]. The rate of DVT and PE in the short term was reported to be less than 1% [22].

The one-year mortality was reported from 15% to 61.9% in the included studies [10-14]. Most patients who received HFNs were multimorbid, thus confounding the mortality rate [10-14]. 

Limitations

This systematic review has several limitations. No randomised controlled trials were available on this topic, and the included studies were all deemed to have moderate to high risk of bias. Moreover, the small sample size and heterogeneous data preclude definitive conclusions. The heterogeneity of outcomes made it difficult to draw clear conclusions. Additionally, not all studies permitted early weight-bearing, undermining the theoretical advantages of HFNs. Functional outcomes were sporadically measured, and follow-up was limited to a maximum of 18 months, precluding long-term outcome analysis. These limitations underscore the need for further high-quality research in this area.

## Conclusions

In conclusion, HFNs remain a viable option for managing open ankle fractures in elderly patients, especially those with low functional demand. Better outcomes were observed when the joint surfaces were not prepared for arthrodesis. HFNs facilitate early weight-bearing, which has several advantages for elderly and frail patients.

## Appendices

Appendix A 

**Table 2:** Showing search terms and results for the OVID Medline database.

Search Terms: OVID Medline	Results
Ankle Joint/ or Ankle/ or Ankle Injuries/ or Ankle Fractures/	38891
"bimalleolar fracture*" or "Trimalleolar fracture*" or "Fibula fracture*" or "Weber fracture*" or "distal tibia fracture*"	1471
1 or 2	39735
Arthrodesis/	10536
"hindfoot nail" or "tibi* calcaneal"	117
4 or 5	10621
3 and 6	2172
limit 7 to english language	1788

S7 AND S8 Limiter English Language	993

Appendix B 

**Table 3:** Search terms and results for Elsevier Embase database

Search Terms : Elsevier Embase	Results
'ankle'/de OR 'ankle joint'/de OR 'ankle fracture'/de OR 'malleolus fracture'/de OR 'bimalleolar fracture'/de OR 'lateral malleolar fracture'/de OR 'medial malleolar fracture'/de	52896
'trimalleolar fracture*' OR 'fibula fracture*' OR 'weber fracture*' OR 'distal tibia fracture*'	4176
#1 OR #2	55804
'arthrodesis'/de OR 'ankle arthrodesis'/de	18910
'hindfoot nail' OR 'tibi* calcaneal'	139
#4 OR #5	18998
#3 AND #6	1920
#3 AND #6 AND [english]/lim	1619

Appendix C 

**Table 4:** Search terms and results for CINAHL database

Search Terms : Ebsco CINAHL	Results
(MH "Ankle") OR (MH "Ankle Injuries") OR (MH "Ankle Fractures") OR (MH "Ankle Dislocation") OR (MH "Ankle Surgery") OR (MH "Ankle Joint")	19528
"bimalleolar fracture*" or "Trimalleolar fracture*" or "Fibula fracture*" or "Weber fracture*" or "distal tibia fracture*"	782
S1 OR S2	19964
(MH "Arthrodesis")	3437
""hindfoot nail" or "tibi* calcaneal""	55
S4 OR S5	3475
S3 AND S6	1010
S7 AND S8 Limiter English Language	993

Appendix D 

**Table 5:** Risk of bias assessment summary table

Study Name and Year published	Authors	Risk of Bias (Newcastle- Ottawa Scale)	Strengths	Weaknesses
Epidemiology & management of complex ankle fractures in the United Kingdom: A multicentre cohort study, 2024 [10]	Stringfellow et al.	Moderate	Multi-centre design Propensity matching help reduce some biases	Retrospective data collection. Lack of a true control group.
Fragility fractures of the ankle in the frail elderly patient treatment with a long calcaneotalotibial nail, 2014 [11]	Al-Nammari et al.	High	Outcomes were assessed adequately	Retrospective design. Unclear representativeness. Lack of control for confounding factors.
Male Sex, Gustillo-Anderson Type III Open Fracture and Definitive External Fixation are Risk Factors for a Return to the OR following the Surgical Management of Geriatric Low Energy Open Ankle Fractures, 2022 [12]	Fourman et al.	Moderate	Adequately represents the target population Follows patients adequately for relevant outcomes	Lack of a comparison group. Lack of control for confounding factors.
The outcomes of the management of complex distal tibia and ankle fractures in elderly with tibiotalocalcaneal nail in a minimum 12-month follow-up period, 2024 [13]	Kotsarinis et al.	Moderate	selection of patients and assessment of outcomes were well defined and executed	Lack of a comparative group. No control cohort group.
The Use of Hindfoot Nails for Elderly Complex Distal Tibial and Ankle Fractures, 2023 [14]	Carin et al.	Moderate	Selection of patients Outcome assessment	Lacks a control group for comparability
